# Personalized cardiovascular risk management for outdoor activities: a digital health application

**DOI:** 10.1093/ehjdh/ztag045

**Published:** 2026-03-10

**Authors:** Marco Vecchiato, Nicola Borasio, Sandro Savino

**Affiliations:** Sports and Exercise Medicine Division, Department of Medicine, University of Padova, Via Giustiniani 2, Padova 35128, Italy; Sports Medicine and Cardiovascular Rehabilitation Unit, Noale Hospital, Venezia 30033, Italy; Department of Theoretical and Applied Sciences, eCampus University, Novedrate, Como 22060, Italy; Sports and Exercise Medicine Division, Department of Medicine, University of Padova, Via Giustiniani 2, Padova 35128, Italy; Institute of Mountain Emergency Medicine, EURAC Research, Viale Druso 1, Bolzano 39100, Italy; Department of Medicine, University of Padova, Via Giustiniani 2, Padova 35128, Italy

**Keywords:** Hiking safety, Tourism, Wearable devices

## Abstract

Hiking is a widely practiced outdoor activity with well-known cardiovascular and mental health benefits. However, its popularity among individuals with chronic conditions and across varying fitness levels raises safety concerns, especially in mountainous environments. Current trail planning tools typically rely on generic metrics, without considering individual cardiovascular risk or functional capacity. To address this gap, a novel digital system named MOVE was developed. Based on a patented algorithm, MOVE integrates user-specific characteristics, such as age, sex, body mass index, physical activity level, cardiovascular risk factors, and chronic conditions, with trail features, such as slope, elevation, and altitude. The system provides estimated cardiorespiratory fitness (eCRF), classifies cardiovascular risk according to ESC guidelines, and generates personalized predictions of hiking time, energy expenditure, heart rate ranges, and relative effort.

Following its public release in late 2024, the app was widely adopted in spring–summer 2025, with over 3000 hikes recorded. Users included individuals with a wide range of eCRF and cardiovascular risk profiles, highlighting MOVE’s potential for real-world applicability. The app was particularly used in collaboration with Alpine tourist regions, supported by trail signage and QR codes. Nearly one-third of hikes were classified as high-effort, mainly in individuals with lower eCRF or greater trail demands.

MOVE represents a promising step towards personalized outdoor physical activity. Future developments include real-time tracking, adaptive feedback, wearable integration, and AI-driven modelling. This approach may enhance safety and accessibility of hiking, particularly for at-risk populations, supporting public health through safe engagement with nature-based exercise.

## Graphical Abstract

**Graphical Abstract:**
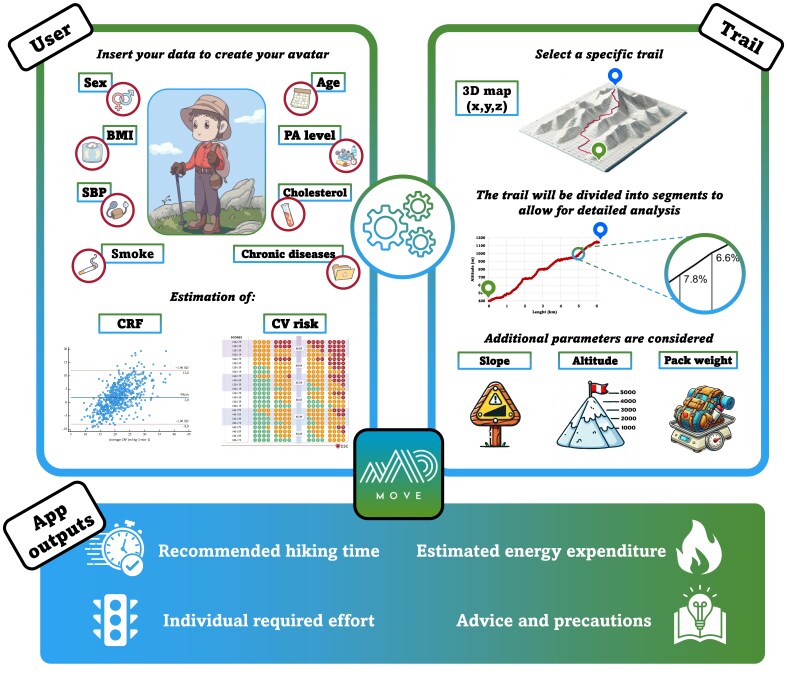
Overview of the MOVE algorithm structure and functioning This figure illustrates the conceptual framework of the MOVE algorithm:User data (left panel): The application collects user-specific information, including mandatory and optional parameters, to estimate cardiovascular risk using the SCORE2/SCORE2-OP algorithms and to estimate cardiorespiratory fitness through validated equations. These values are then used to define individualized exercise thresholds according to the user’s estimated cardiovascular risk profile.Trail characteristics (right panel): Hiking paths are reconstructed from 3D GPX-derived maps and represented in two dimensions. The trail is segmented into multiple short fractions with known length and slope. For each segment, local slope, altitude, and carried load (e.g. backpack weight) are integrated to calculate the overall hiking effort and energy cost.App outputs (bottom panel): Based on user data and trail-specific parameters, MOVE provides four individualized predicted main outputs: hiking time, physical effort, energy expenditure, and safety recommendations according to cardiovascular risk and chronic diseases.

## Introduction

Hiking is an increasingly popular outdoor recreational activity, especially during the summer season, with increasing accessibility due to available technical equipment and infrastructures. Outdoor activities are now practiced by a broad population, including individuals with chronic conditions or those seeking outdoor activities for their physical and mental well-being. Benefits include improved functional capacity, also affecting cardiorespiratory fitness, coordination, and reducing stress levels.

However, outdoor activities inherently carry risks. Following the COVID-19 pandemic, there has been a substantial rise in nature-based tourism all around the world with a parallel increase in accidents, primarily due to limited awareness of environment-specific risks.^1^ While climbing or alpine mountaineering could be expected as high-risk sports, hiking ranks first among outdoor activities requiring rescue operations, accounting for about 50% of all mountain rescue calls.^2^ The most frequent causes include falls, slips, fatigue, and acute medical events, often due to an underestimation of the required effort or inappropriate planning and trail selection. Notably, sudden cardiac death remains the leading cause of death among men over 34 years of age during mountain excursions.^3^

Although traditional hiking signage and online platforms provide useful data on trail length, altitude, elevation profile, and technical difficulty, they generally do not consider individual pathophysiological characteristics and lifestyle. Hiking times are typically estimated based on average users, with no personalized adjustment for physical activity and fitness level, health status, or cardiovascular risk profile. Despite the availability of national and international standards for trail classification (e.g. Swiss and Italian Alpine associations), most hikers must still rely on generic information. Moreover, such route signage typically estimates that an average person gains about 350 m per hour of ascent and 500 m per hour of descent, also considering changes in altitude. These values are helpful but fail to account for the high inter-individual variability regarding cardiorespiratory fitness, exercise tolerance, and relative cardiovascular demand.

## MOVE: a novel digital tool for risk-stratified and fitness-based hiking guidance

In recent years, the fitness and digital health sectors have increasingly incorporated individual physiological and cardiovascular data into physical activity and exercise planning. Numerous commercial applications and devices now provide training recommendations tailored to parameters, such as age, heart rate, or estimated cardiorespiratory fitness (eCRF) reflecting a broader trend towards data-driven personalization in physical activity and sports.^4^ This approach appears particularly relevant for outdoor activities such as hiking, when considering the heterogeneous fitness levels and clinical conditions for a potentially demanding cardiorespiratory effort in an uncontrolled mountain environment. Nonetheless, most route-planning tools currently available on the market for outdoor use, continue to rely on generic metrics, without accounting for individual health status or cardiovascular risk.^5^

To address this implementation gap and to promote the health benefits for outdoor physical activities in natural environments, a patented algorithm has been developed integrating user-specific characteristics, such as age, sex, body mass index (BMI), physical activity level, cardiovascular risk factors, and possible chronic conditions, into the planning of hiking itineraries (**Graphical Abstract**). The system employs scientifically validated equations to estimate eCRF and uses cardiovascular risk classification according to the most recent ESC guidelines.^6,7^ The algorithm dynamically selects the most appropriate eCRF equation according to the individual user profile (age, sex, BMI, and physical activity level), based on reported performance metrics of validated models. eCRF is used exclusively for relative effort stratification and preventive guidance, and not for clinical fitness diagnosis or performance prescription. Based on these outputs, the system defines a recommended physical effort threshold, expressed as a percentage of eCRF, in accordance with the ESC guidelines on physical activity and cardiovascular disease prevention.^8^

This digital model has been implemented in a prototype application, called MOVE (publicly available as MOVE – hike and bike), which analyses trail features such as altitude, elevation change and slope in association with the user profile to provide personalized estimates of recommended hiking time, energy expenditure, heart rate intensity ranges, and relative physical effort.^9^ These outputs support informed trail selection based on an individual’s functional capacity and risk profile.^5^

Besides providing specific qualitative recommendations for different pathologies, the algorithm also considers other real-world variables such as load carriage and altitude to refine eCRF estimates and effort prediction. Carrying a backpack increases oxygen consumption, thus significantly affecting aerobic capacity with an impact of about ∼20% per 10% additional load relative to body weight. The algorithm takes this information and adjusts the outputs based on backpack weight. Similarly, altitude exposure reduces aerobic capacity by ∼1% per 100 m above 1500 m; thus, an approximately 20% reduction is expected at 3500 m. These adjustments aim to provide more realistic and tailored estimates aligned with the physiological demands of hiking.^10^

## Real-world implementation: participant characteristics and trail data

Since its public release on online platforms in autumn 2024, MOVE has been broadly adopted, particularly during the spring-summer 2025 season, following partnerships with selected Italian Alpine tourist regions. Dedicated trail signage was installed, including vertical QR codes linking to the app interface. Between April and September 2025, 3004 hiking records were collected through the app, providing insight into its practical application and user demographics (***Table 1***).

**Table 1:** Demographic and clinical characteristics of MOVE app users and selected hiking trail features

Variables		All		Women		Men	
		*n* = 3004		*n* = 832		*n* = 2172	
**Age**	**Years**	**Mean**	**SD**	**Mean**	**SD**	**Mean**	**SD**
		48.17	14.64	44.60	12.15	49.54	15.27
		***n***	**%**	***n***	**%**	***n***	**%**
	**< 40**	884	29	283	34	601	28
	**40 -55**	889	30	337	41	552	25
	**55–70**	1058	35	209	25	849	39
	**> 70**	173	6	3	0	170	8
**BMI**	**kg/m^2^**	**Mean**	**SD**	**Mean**	**SD**	**Mean**	**SD**
		23.98	3.29	23.01	3.78	24.35	3.01
		***n***	**%**	***n***	**%**	***n***	**%**
	**Underweight**	47	2	41	5	6	0.3
	**Normal weight**	2057	68	610	73	1447	67
	**Overweight**	752	25	132	16	620	29
	**Obesity**	148	5	49	6	99	4
**eCRF**	**mL/kg/min**	**Mean**	**SD**	**Mean**	**SD**	**Mean**	**SD**
		37.13	8.13	32.83	7.49	39.07	7.65
**Blood Pressure**		***n***	**%**	***n***	**%**	***n***	**%**
	**Normal**	1871	62	567	68	1304	60
	**On target with therapy**	605	20	97	12	508	23
	**Elevated despite therapy**	11	0.4	0	0.0	11	1
	**Elevated**	89	3	2	0.2	87	4
	**Not known**	428	14	166	20	262	12
**Cholesterol**	**Normal**	1778	59	500	60	1278	59
	**On target with therapy**	277	9	63	8	214	10
	**Elevated despite therapy**	19	1	12	1	7	0.3
	**Elevated**	299	10	10	1	289	13
	**Not known**	631	21	247	30	384	18
**Smoking**	**Yes**	428	14	51	6	377	17
	**No**	2419	81	719	86	1700	78
	**Not declared**	157	5	62	7	95	4
**CV risk score**	**Low-moderate risk**	967	32	296	36	671	31
	**High risk**	324	11	83	10	241	11
	**Very high**	184	6	49	6	135	6
	**<40 y with high risk**	15	0.5	0	0	15	1
	**Not applicable**	1514	50	404	49	1110	51
**Physical activity** **level**	**Sedentary**	1065	35	292	35	773	36
	**< 150 min/week**	805	27	172	21	633	29
	**150–300 min/week**	544	18	175	21	369	17
	**> 300 min/week**	590	20	193	23	397	18
**Diseases**	**Not declared**	490	16	35	4	455	21
	**No**	2227	74	710	85	1517	70
	**Musculoskeletal**	49	2	21	3	28	1
	**Asthma**	79	3	38	5	41	2
	**Diabetes**	67	2	1	0.1	66	3
	**Heart diseases** ^a^	79	3	29	3	50	2
	**Kidney**	10	0.3	1	0.1	9	0.4
	**Neurological**	7	0.2	2	0.2	5	0.2
	**Pulmonary** **(not asthma)**	7	0.2	1	0.1	6	0.3
	**Transplant**	2	0.1	1	0.1	1	0.0
**Trail Characteristic**		**Mean**	**SD**	**Mean**	**SD**	**Mean**	**SD**
	**Length (m)**	10008	7972	9483	6690	10205	8394
	**Elevation Gain (m)**	804	609	699	536	844	630
		***n***	**%**	***n***	**%**	***n***	**%**
**Trail Technical Difficulty**	**T—Tourist**	740	25	282	34	458	21
	**E—Hiking**	1712	57	394	47	1318	61
	**EE—Expert Hiking**	552	18	156	19	396	18
**Predicted physical effort**	**Low**	1434	48	450	54	984	45
	**Medium**	625	21	210	25	415	19
	**High**	945	31	172	21	773	36

BMI, body mass index; BP, blood pressure; eCRF, estimated cardiorespiratory fitness; HR, heart rate; MET, metabolic equivalent of task; E, Hiking (Escursionistico—CAI classification); EE, Expert Hiking (Escursionisti Esperti—CAI classification).

^a^Heart diseases do not include arterial hypertension.

The cohort included 2172 men and 832 women, with a mean age of 48.17 ± 14.64 years. Most users were aged between 40 and 70 years (65%) and had a normal BMI (23.98 ± 3.29 kg/m^2^). Based on self-reported values and SCORE2/SCORE2-OP classifications, the cardiovascular risk profile included 967 individuals at low-to-moderate risk (32%), 324 at high risk (11%), and 184 at very high risk (6%), while 15 users <40 years old showed high risk based on medical history.

The mean eCRF was 37.1 ± 8.1 mL/kg/min, with significantly lower values in women. Blood pressure and cholesterol levels were reported as normal or well controlled in over 70% of participants, though 14% and 21% of users were unaware of their respective values. Notably, 14% were active smokers.

The most common trails selected had a mean length of ∼10 km and an average elevation gain of ∼800 m, with technical classifications of E (Hiking, 57%) and EE (Expert Hiking, 18%) being the most represented. Despite a wide range of physical fitness and clinical status, 31% of the hikes were classified by the algorithm as high effort, particularly among those with lower eCRF and/or greater elevation gain.

## Limitations and future directions

This study does not report clinical outcomes such as adverse cardiovascular events or demonstrated safety improvements. The analysis is descriptive and exploratory, focusing on feasibility, implementation, and real-world usage patterns. Prospective studies evaluating clinical outcomes and safety endpoints are planned.

A key limitation of the current dataset is the reliance on self-reported health and lifestyle data, including blood pressure, cholesterol levels, and physical activity habits. As expected in a non-clinical, population-based setting, a substantial proportion of users were unable to report exact values for these parameters. To mitigate this issue, health variables were collected using predefined categories rather than continuous values, and missing information was explicitly classified as ‘unknown’. In cases where cardiovascular risk could not be calculated using risk charts due to incomplete data, a conservative risk classification strategy was adopted, assigning the highest plausible risk category based on the available information in order to avoid risk underestimation. This precautionary approach prioritizes user safety but may lead to overestimation of cardiovascular risk in some individuals.

Although directly measured CRF values are not available in the MOVE real-world dataset, we previously conducted controlled laboratory and field-based physiological testing using CPET and portable breath-by-breath gas analysis during hiking to objectively characterize the cardiorespiratory demands of hiking and the inter-individual variability in relative exercise intensity.^11^ These findings support the physiological rationale underlying MOVE’s effort modelling approach. Dedicated studies directly comparing eCRF and directly measured CRF values are planned as part of future research.

The current application processes the GPX file of the selected route, considering the associated user parameters and provides an overall trail-level assessment. However, future developments will offer more precise analyses, through segment-based breakdowns of trails and real-time tracking of the user position and speed, with adaptive recommendations and timely feedback along the trail.

Processing current and past tracking data of a single user with machine learning algorithms will allow better predictions of the user’s fitness level and expected performance; on the other hand, multi user data will be used to detect and offset errors in the route (e.g. accounting for a constant or seasonal bias due to the terrain type) to improve the algorithm outputs. Integration with wearable devices is expected to further improve the accuracy of the system due to live monitoring and processing of physiological data such as step cadence, heart rate, ventilation or oxygen saturation. In this evolving digital health landscape, applications designed for outdoor activities should move towards evidence-based personalization, particularly for populations at increased cardiovascular risk. The incorporation of artificial intelligence could further enhance these systems, enabling methods for user profiling, real-time data analysis, predictive modelling, and tailored feedback based on user trends and biometric feedback loops.

On the other hand, the growing presence of digital platforms, outdoor-specific social media and route-sharing websites has increased visibility and accessibility to mountain tourism, without adjusting for the related risks with specific target initiatives. Non-personalized recommendations may inadvertently promote overexertion or unsafe behaviour, particularly among less experienced or higher-risk individuals. The potential for these platforms to support individualized planning based on fitness and risk profiling represents a valuable direction and opportunity for public health initiatives and health tourism by promoting safe and salutary physical activity in nature mountain environment.

## Conclusions

While outdoor activities like hiking offer significant cardiovascular and psychological benefits, they must be approached with a greater degree of individualized planning, especially as participation expands across age and risk spectrums. Digital health tools integrating cardiovascular risk assessment, pathophysiological profiling, physical activity, and fitness evaluations as well as hiking trail analysis may serve as valuable allies in this effort. A patented algorithm currently implemented in an early-phase digital application suggests the feasibility and first promising outcomes of this approach. In the near future, the incorporation of artificial intelligence and wearable technology may further support hikers in making informed, health-conscious and thus safe decisions before and during such outdoor activities.

### Ethical considerations

No identifiable personal data were collected through the *MOVE - hike and bike* application. All data analysed in this study were fully anonymized and aggregated. According to European data protection regulations, the analysis of fully anonymized data does not involve the processing of personal data. Therefore, in accordance with institutional policies, this study did not require formal ethics committee approval.
